# 

**Published:** 1966-01

**Authors:** 


NATIONAL LIBRARY OF MEDICINE

Jan 66

RECD. FEB 23 1966

ASG JTC

81-299 VOL-ISSUE

INDEXER

January 1966

### Medico-Chirurgical Journal

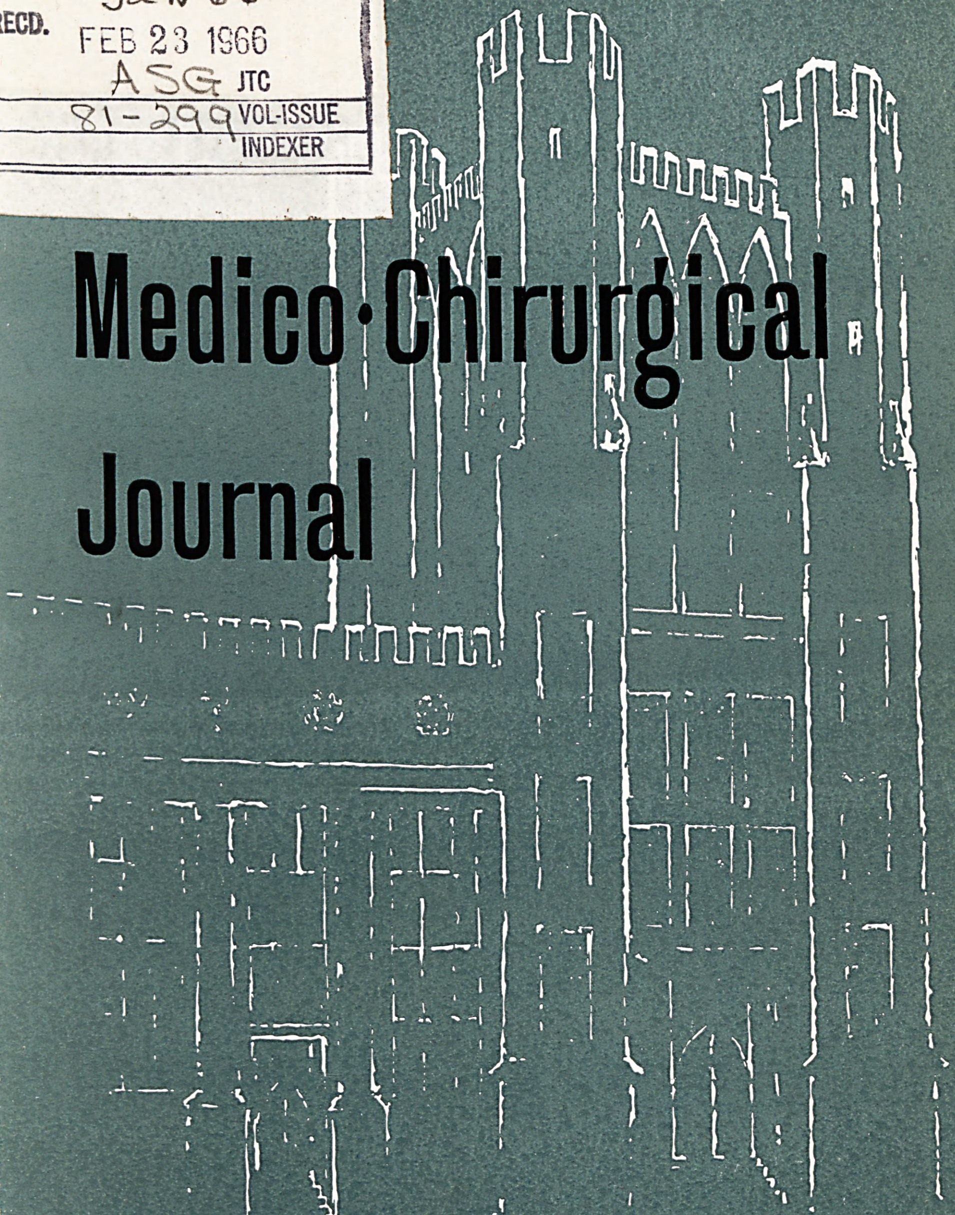

INCORPORATING THE MEDICAL JOURNAL OF THE SOUTH WEST
Vol 81 (i) No 299

